# Supplementary material for: Deep CO2 in the end-Triassic Central Atlantic Magmatic Province
Source: Nat Commun. 2020 Apr 7;11:1670. doi: 10.1038/s41467-020-15325-6 (PMC7138847; doi:10.1038/s41467-020-15325-6)
Supplement: Supplementary file 1 — Supplementary Information [file 41467_2020_15325_MOESM1_ESM.pdf]

Supplementary Information

Deep CO_2 in the end-Triassic Central Atlantic Magmatic Province

Capriolo et al.

Supplementary Notes

Supplementary Note 1. Most analysed bubbles from MIs are characterized by the presence of CO_2 or, less frequently, of elemental carbon and no detectable amounts of any other investigated volatile phase, such as H_2O or SO_2. The large variability in volume and number of bubbles per MI reveals heterogeneous entrapment of MIs[1,2]. In other words, the bubbles are interpreted as gas exsolution bubbles of a CO_2-rich fluid phase, likely exsolved from the silicate melt prior to, or during, their entrapment. Within gas exsolution bubbles, elemental carbon and CO_2 are never present together in the same rock sample. Elemental carbon is not optically detectable and therefore it is probably present as a thin film coating the inner spherical surface of the bubbles. Moreover, it displays different degrees of crystallinity, from amorphous carbon to disordered graphite, occurring also together in the same rock sample. The elemental carbon likely forms from CO_2 upon cooling and reduction of the gas-bearing bubbles. The change in oxidation state may be due, for instance, to a diffusive loss of oxygen from the bubbles to the melt, when the latter crystallizes oxides (e.g., magnetite) during cooling. Notably, small magnetite crystals have been detected by confocal Raman microspectroscopy within MIs. By contrast, it is unlikely that elemental carbon was directly exsolved from the silicate melt. Graphite saturation from a basaltic melt is only possible at reduced conditions, for instance resulting from assimilation of organic matter-rich sediments[3]. However, the elemental carbon is within the bubbles and not within the MIs glass, indicating that it formed from a gas. When melt is trapped as MIs without gas exsolution bubbles, MIs usually develop a single shrinkage bubble per inclusion as result of the differential contraction of the glass compared to the host crystalline phase during cooling[4,5]. The formation of such bubbles results in a strong depletion of CO_2 from the trapped melt, whereas H_2O remains into the melt[2]. When one or more gas exsolution bubbles are trapped along with melt to form the MIs, they may be able to accommodate the post-entrapment shrinkage. The migration of CO_2 from the melt to the bubbles explains, at least partially, the low (< 100 ppm) CO_2 concentrations in the MIs glass (Supplementary Tab. 5). Some bubbles, from both single- and multi-bubble MIs, appear to be empty. These are interpreted as emptied gas exsolution bubbles or emptied shrinkage bubbles (only in the case of single-bubble MIs), where the leakage or total loss of carbon species was likely due to mechanical micro-fracturing (i.e., micro-cracking). The expansion (i.e., volume increase) of bubbles during cooling and decompression may also support the relatively low values of density of CO_2 within all the analysed bubbles, whose presence indicates that melt was saturated in CO_2 prior to the entrapment. The volatile-saturated melt and the volatiles within MIs may be cogenetic (i.e., entrapment of melt and volatiles immediately after, or during, gas exsolution), or may derive from distinct sources (i.e., entrapment of melt and volatiles exsolved from deeper magmas, or degassed and fluxed from the intruded crustal rocks). In both cases, the volatile-saturated melt plus gaseous bubbles were entrapped within interstices of a growing crystal mush during magma rise, forming heterogeneous MIs (i.e., displaying different glass/bubble ratios). On the contrary, gas exsolution did not occur within MIs after melt entrapment, because the trapping of an originally gas bubble-free melt would have produced homogeneous MIs (i.e., displaying very similar glass/bubble ratios). Instead, the investigated MIs show variable volume fractions of bubbles, indicating that these MIs trapped both melt and fluid (i.e., bubbles were randomly entrained during MI formation). The entrapment of CO_2-bearing bubbles present in the melt suggests rapid growth of the clinopyroxene crystals, that was likely triggered by a sudden decrease of the pressure associated with volatile exsolution, or by a progressive increase of the liquidus temperature due to the CO_2-rich fluids fluxing, which reduces the chemical potential of H_2O in the system, promoting

crystallization[6-8]. Moreover, the widespread presence of bubbles within MIs and their only occasional coalescence reveal that in most cases the melt viscosity prevented the total coalescence of bubbles in a single bubble within the entrapped melt. These features suggest that the entrapped melt was viscous (i.e., relatively cold and differentiated), consistently with a rapid cooling of the MIs.

Supplementary Note 2. Crystallization temperature and pressure of the host clinopyroxene crystals can be calculated from mineral compositions (Supplementary Tab. 6) using geothermobarometers developed for magmatic systems[9,10]. The geothermobarometer based on the equilibrium between two distinct pyroxenes[10] was applied to sample NEW31, where augite and pigeonite coexist in textural and chemical equilibrium. For this sample, the calculated temperature is 1308 ± 45 °C, and the calculated pressure is 0.8 ± 0.4 GPa, corresponding to lower-middle crustal depths (ca. 27 ± 13 km). The geothermobarometer based on the equilibrium between clinopyroxene and a magmatic liquid[9] was taken into account for samples AL14, AN18, AN137A, NEW31, NS9 and NS12. Using this geothermobarometer, both the MIs glass and the whole rock may be considered as possible proxies for the original magmatic liquid composition. However, in terms of Fe/Mg K_d and predicted/observed clinopyroxene components, MIs glass does not appear to be in equilibrium with the host clinopyroxene crystals. Instead, the whole rocks appear to be in chemical equilibrium with the host clinopyroxene crystals for samples AN18, NEW31, NS9 and NS12. For all these samples, the calculated temperature ranges from 1150 to 1230 °C (uncertainty ± 27 °C), and the calculated pressure ranges from 0.1 to 0.7 GPa (uncertainty ± 0.2 GPa), corresponding to middle crustal depths (on average ca. 12 ± 7 km; Supplementary Fig. 7). All the calculated temperature and pressure values suggest that most of the host clinopyroxene crystallized within the middle crust, even if some crystals may have formed at shallower depths. The obtained crystallization pressures are consistent with those calculated for augite crystallization from throughout the CAMP, including Portugal, Morocco, USA, and Canada, ranging from 0.2 to 0.8 GPa (uncertainty ± 0.2 GPa)[11-13], and are also comparable to those estimated for CAMP layered mafic intrusions, such as the Freetown Complex, Sierra Leone[14]. All these data suggest that the crystallization of clinopyroxene in CAMP basalts occurred at various depths ranging from the deep to the relatively shallow crust, consistent with the existence of a transcrustal magmatic plumbing scenario[15,16]. The host crystals are usually part of glomerocrystic clinopyroxene aggregates, and the shapes of MIs are generally irregular, suggesting that the melt was entrapped in crystal interstices during rapid crystal growth. These glomerocrysts can be interpreted as clots of partially crystallized mineral aggregates. In fact, mush reservoirs within the deep roots of magmatic plumbing systems commonly consist of closely packed crystals, forming a porous and permeable framework, with interstitial melt[15,17]. Such interstitial melt is likely to be more evolved, and also enriched in volatiles, than a typical basaltic melt, probably representing the residuum after formation of the crystal mush. Crystalline mushes may form at various depths within the magmatic plumbing system of LIP basalts, from the deep to the shallow crust, where the magma is forced to stall due to negative buoyancy barriers[15,17]. Hence, the crystalline mush reservoir of growing crystals, infiltrated by an ascending interstitial basaltic melt, implies a condition of general equilibrium between growing crystals (with slightly different compositions, as function of depth) and the percolating melt (with nearly constant composition, due to recharging pulses from the source). This scenario is also consistent with the chemical equilibrium observed between the host clinopyroxene crystals and the whole rock.

Supplementary Note 3. The different partitioning of CO_2 and H_2O between glass and bubbles within MIs, observed through confocal Raman microspectroscopy and NanoSIMS, reveals that CO_2 is mostly present in the bubbles, and H_2O is mostly present in the glass. In particular, the CO_2/H_2O ratio in the glass is approximately 0.01, and no H_2O but only CO_2 was detected through confocal Raman microspectroscopy in the bubbles. Nevertheless, for the observed MIs glass composition and the measured weight fraction of dissolved H_2O and CO_2 within MIs glass, at shallow depths the coexisting fluid phase (calculated using the model of ref. 18) is expected to contain H_2O, that was never detected in the bubbles. Diffusive re-equilibration is a common post-entrapment process affecting MIs, and especially involving volatiles, such as H^+ and, to a lesser extent, CO_2[19,20]. In more detail, despite different diffusivities, CO_2 preferentially diffuses from the glass into the bubbles (once bubbles have accommodated the post-entrapment shrinkage), and H^+ preferentially diffuses from the MIs outwards, through the host crystals, resulting in a loss of H_2O[5,21,22]. This may suggest a post-entrapment diffusive re-equilibration within MIs for both CO_2 and H_2O, which involved a depletion in CO_2 of the glass, and a depletion in H_2O of both glass and bubbles. However, despite this post-entrapment diffusive re-equilibration, the constant presence of CO_2 (or elemental carbon) and absence of H_2O within the bubbles indicate that the primary composition of the fluid phase forming the bubbles was CO_2-rich and H_2O-poor.

Supplementary Figures

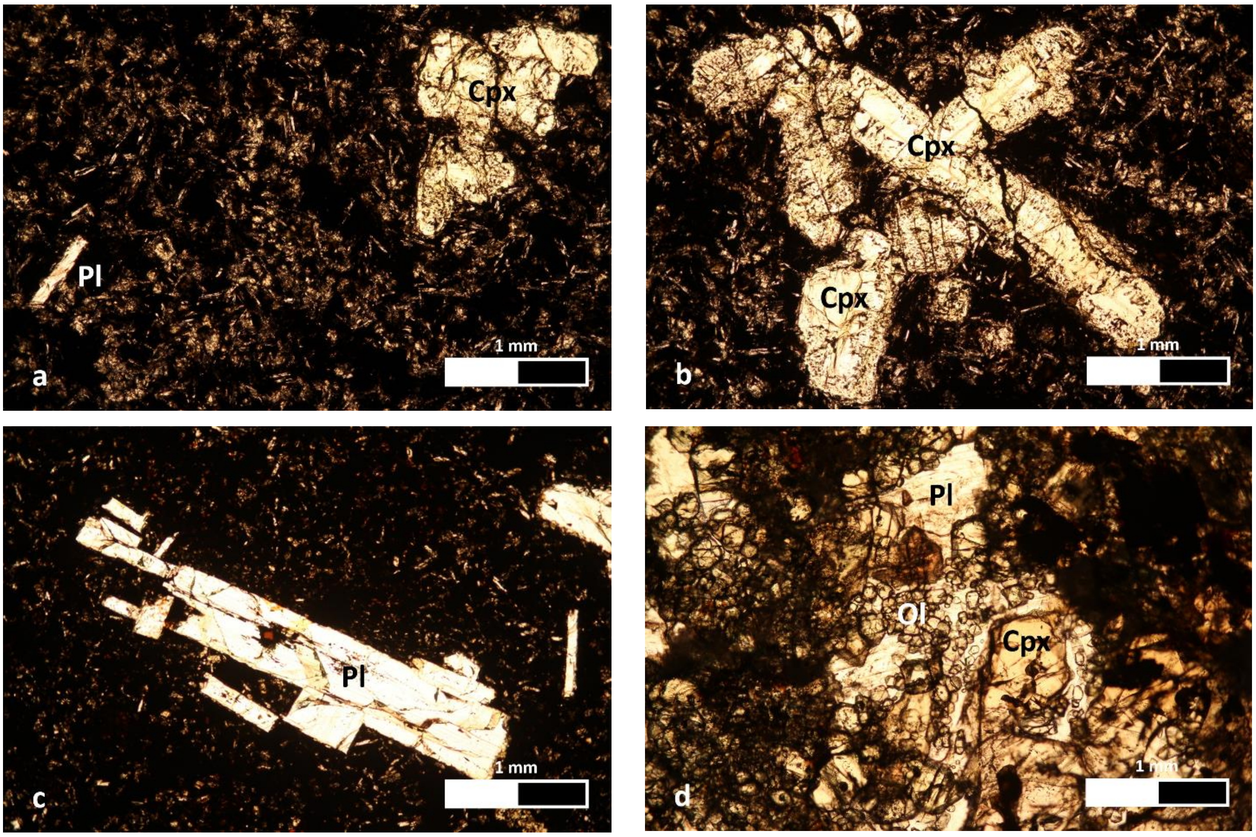

Supplementary Figure 1 Representative analysed samples at transmitted light optical microscopy. a) Porphyritic and microcrystalline texture with phenocrysts of clinopyroxene and plagioclase (Cpx and Pl; sample NEW31, New Jersey, USA). b) Glomerocrystic aggregate of clinopyroxene (Cpx; sample NEW31, New Jersey, USA). c) Large phenocryst of plagioclase (Pl; sample AN156A, Morocco). d) Crystals of plagioclase, clinopyroxene and well-preserved olivine (Pl, Cpx and Ol; sample NEW136B, New Jersey, USA).

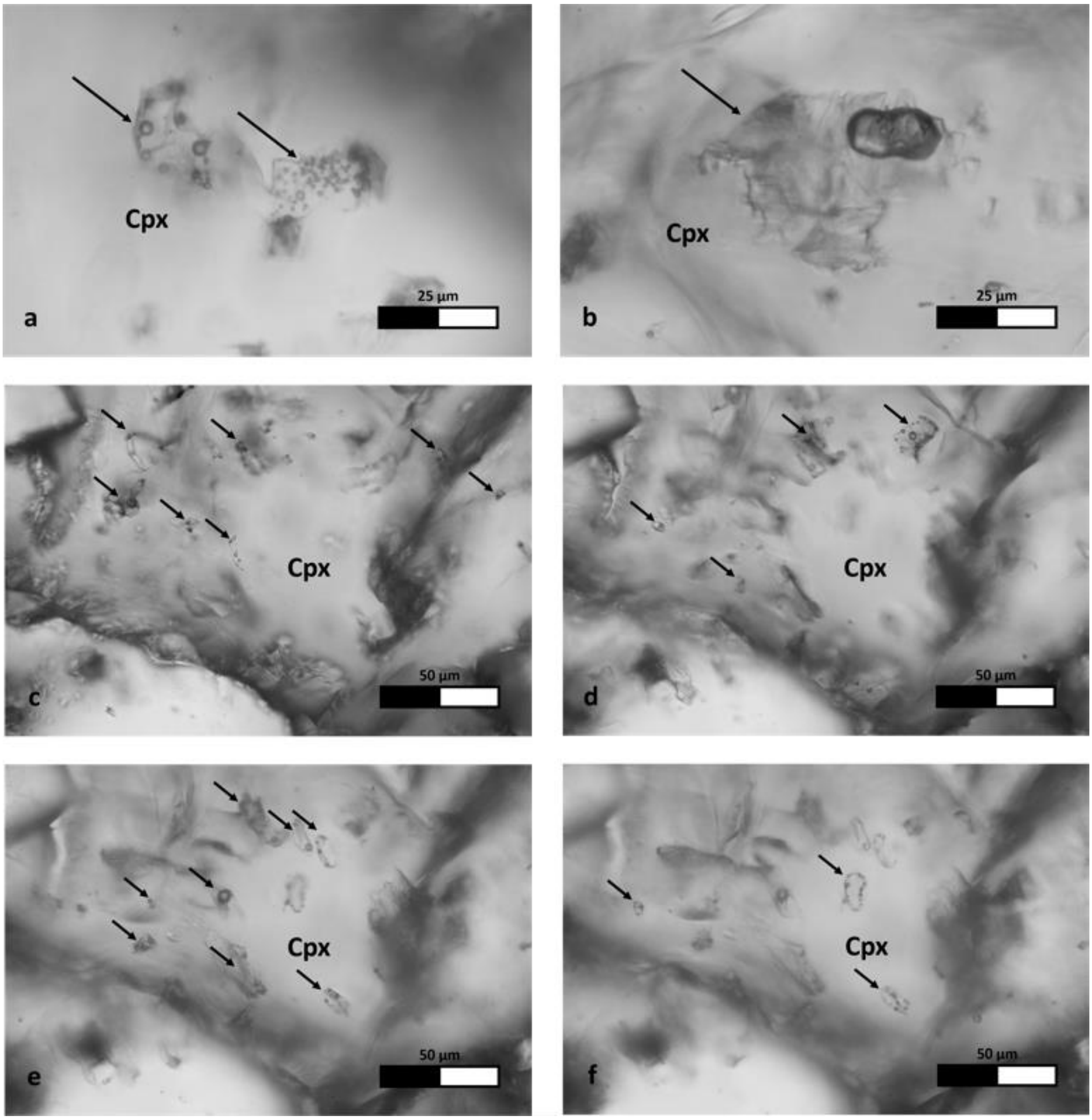

Supplementary Figure 2 Representative single- and multi-bubble melt inclusions at transmitted light optical microscopy. The black arrows indicate the bubble-bearing melt inclusions. a) Multi-bubble MIs characterized by a highly variable range in bubble size, hosted in augitic clinopyroxene (Cpx; sample NS12, Nova Scotia, Canada). b) Coalescent bubbles within MI, hosted in augitic clinopyroxene (Cpx; sample AN18, Morocco). c-f) Single- and multi-bubble MIs hosted in augitic clinopyroxene (Cpx) at increasing depth in the same crystalline aggregate, displaying different ratios between the volume of glass and the volume/number of bubbles (sample NS9, Nova Scotia, Canada).

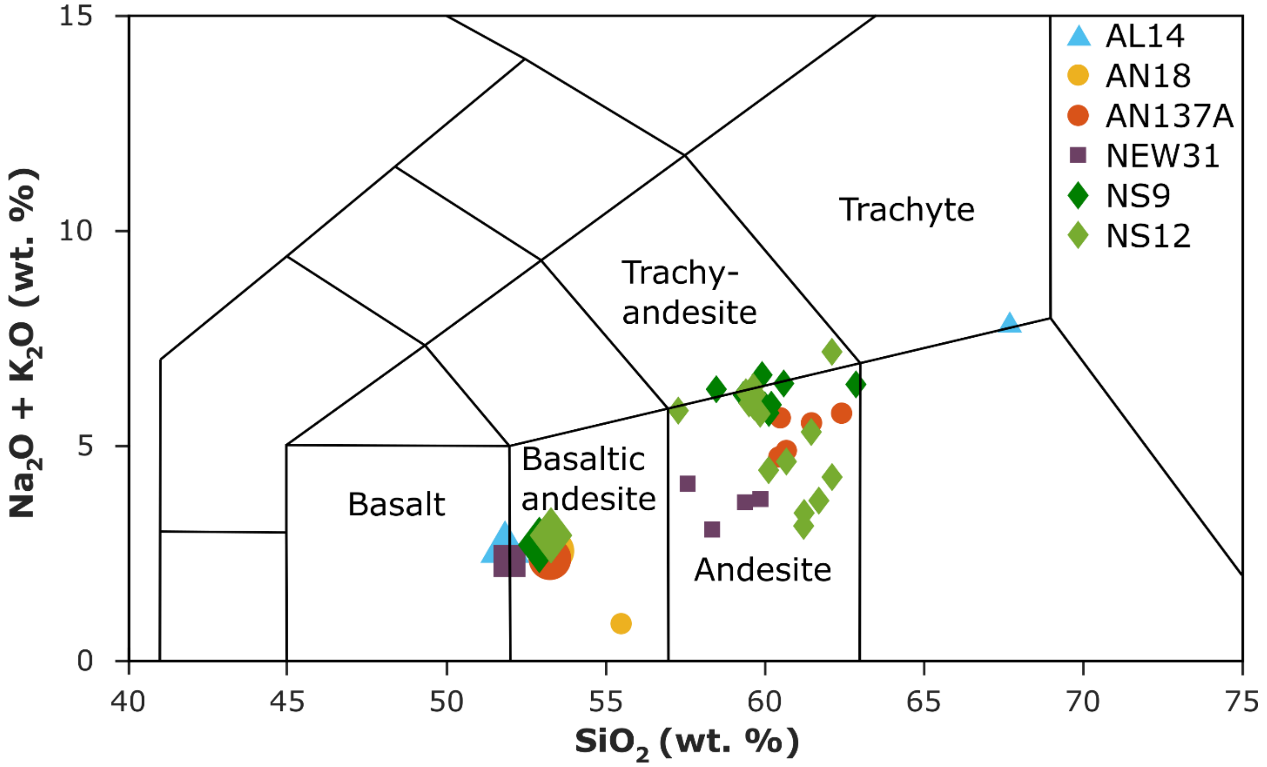

Supplementary Figure 3 MIs glass and whole rock classification. The small symbols indicate the MIs glass composition, and the large symbols indicate the whole rock composition on the total alkali-silica (TAS) classification diagram[23]. All the data are recast to anhydrous values and recalculated to 100 wt. %. The whole rock compositions are from refs. 11, 12 and 13.

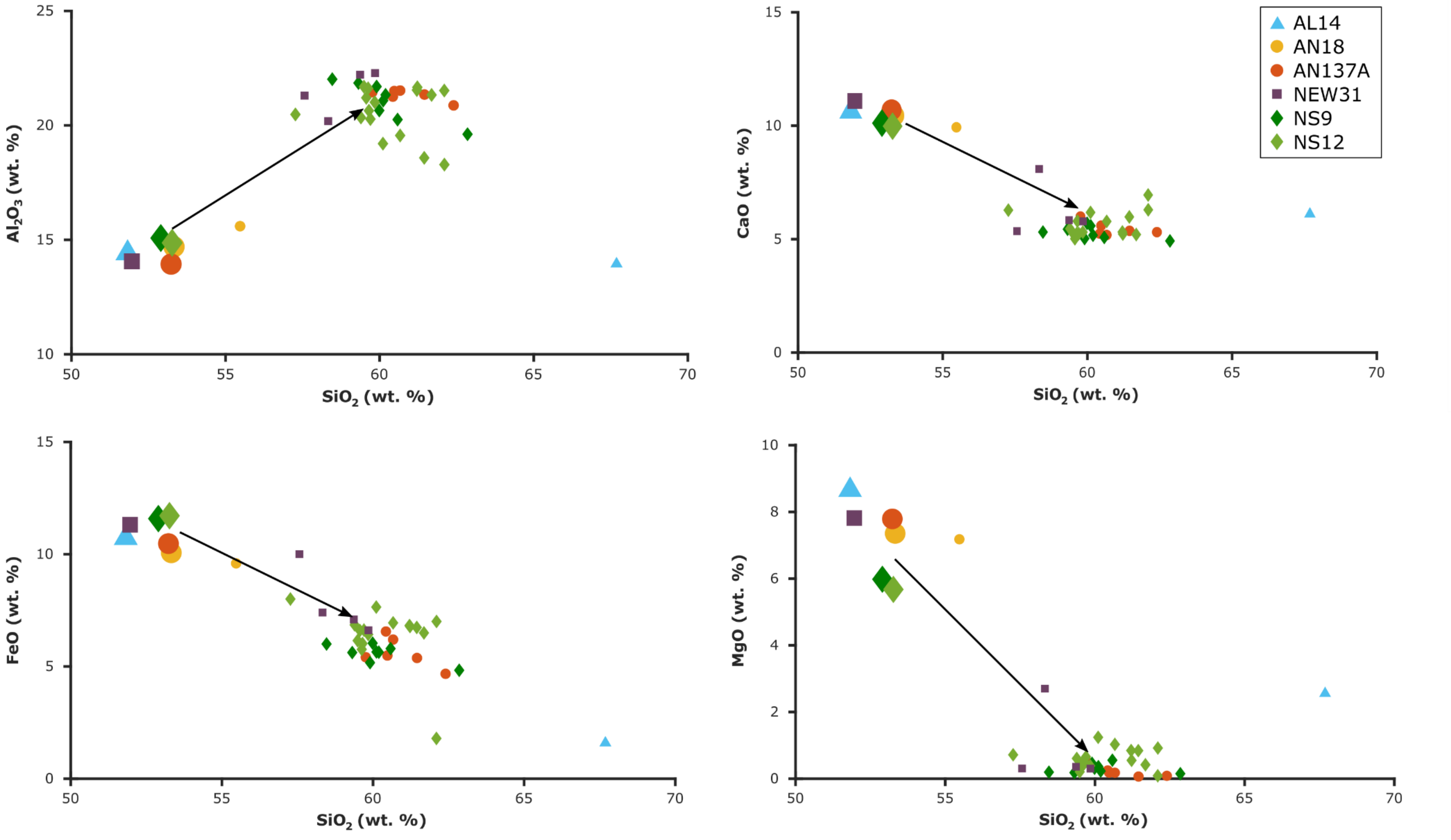

Supplementary Figure 4 MIs glass and whole rock composition. The small symbols indicate the MIs glass composition, and the large symbols indicate the whole rock composition on the plots of Al_2O_3, CaO, FeO, and MgO versus SiO_2 contents. The black arrows indicate the variation of composition due to ca. 55 % fractional crystallization of augitic clinopyroxene (39 %), plagioclase (11 %) and magnetite (5 %) as calculated by mass balance. All the data are recast to anhydrous values and recalculated to 100 wt. %. The whole rock compositions are from refs. 11, 12 and 13.

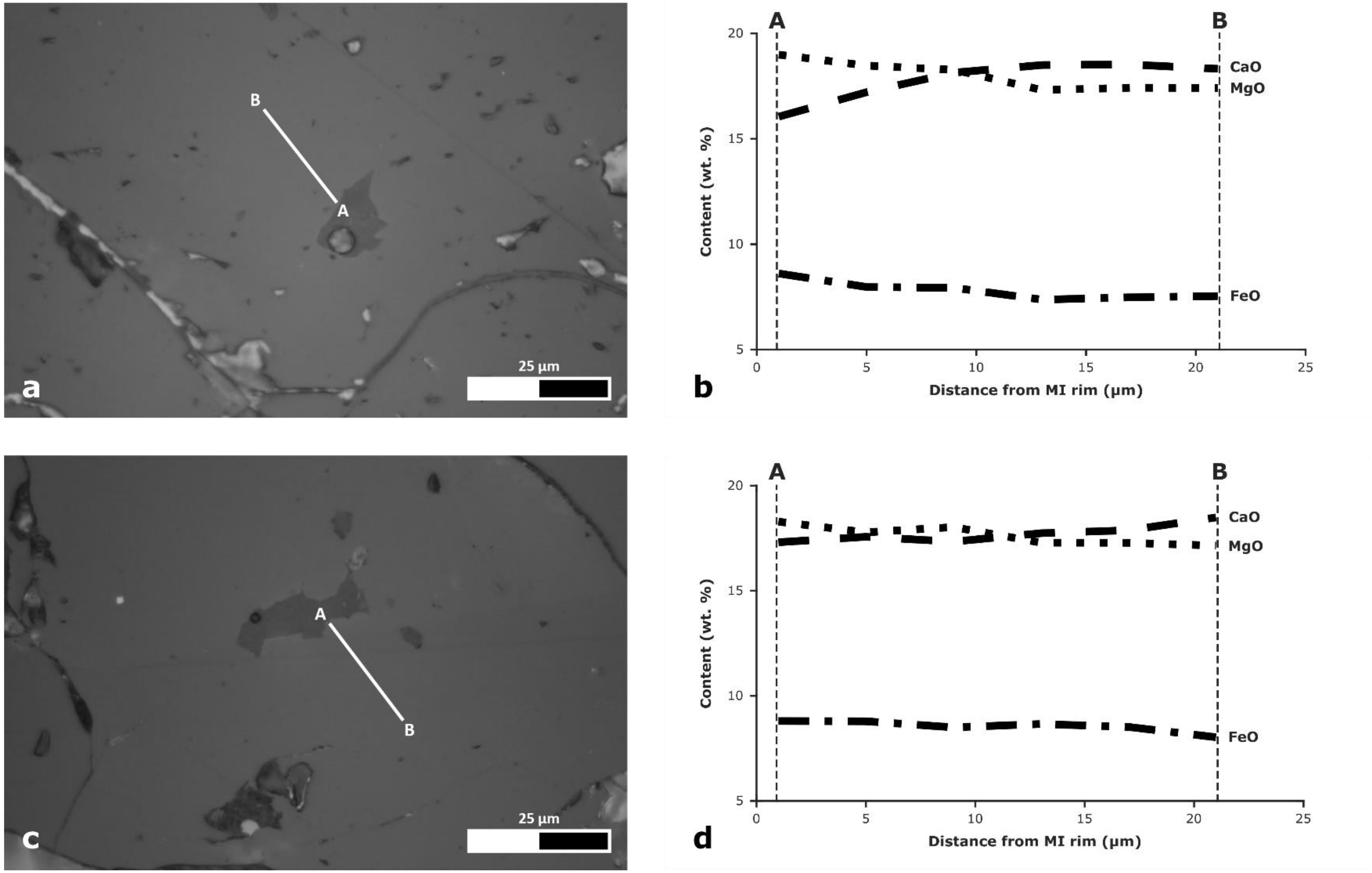

Supplementary Figure 5 Electron microprobe transects in glomerocrystic clinopyroxene aggregates. Left column: reflected light photomicrographs at optical microscope of the analysed transects (a: sample AN137A, Morocco; c: sample NS12, Nova Scotia, Canada). The exposed MIs, pointed out by cut bubbles, are darker than the host clinopyroxene. Right column: plot of CaO, FeO and MgO variations along clinopyroxene transects from the rim of MI to the core of the host mineral (b and d). In both transects, a decrease in CaO content and a slight increase in both MgO and FeO content close to the MI rims are evident.

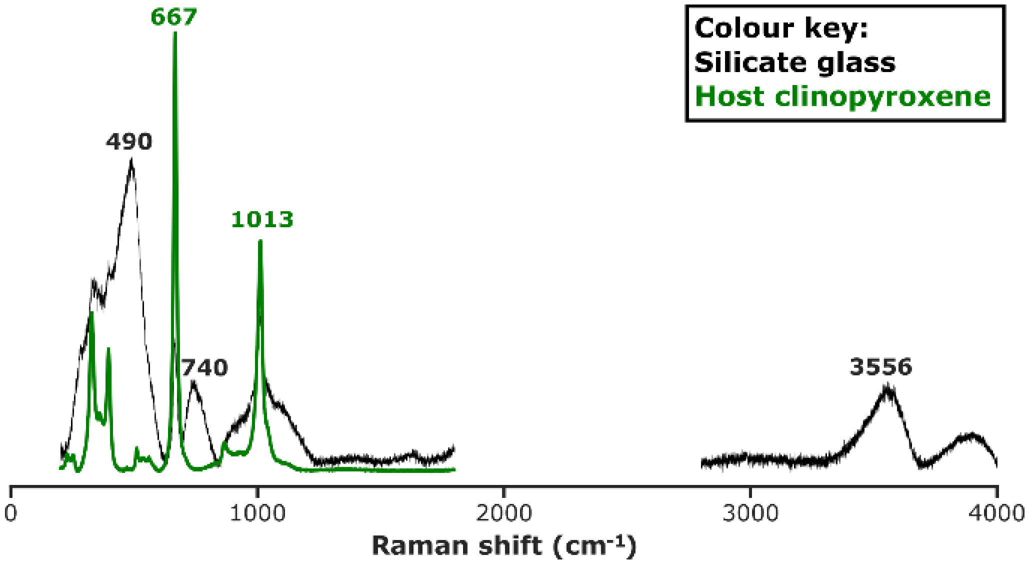

Supplementary Figure 6 Comparison between Raman spectra of the glass in an exposed single-bubble MI and its host clinopyroxene. The Raman spectrum of the glass, even if weak, is characterized by two broad bands, approximately at ~500 cm^{-1} and ~750 cm^{-1}, and by the H_2O band, at ~3600 cm^{-1}. All other features of this Raman spectrum are inherited from the Raman signal of the surrounding host clinopyroxene, as can be seen from the coincidence of the peaks position. These Raman spectra were acquired on sample AN39 (Morocco).

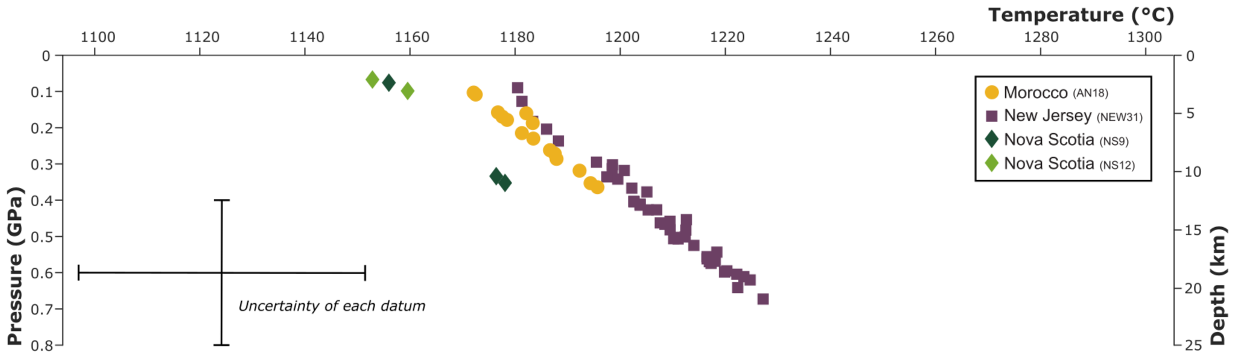

Supplementary Figure 7 Geothermobarometry on host clinopyroxene. Clinopyroxene crystallization pressures and temperatures have been calculated combining analysed clinopyroxene and whole rock compositions, using ref. 9. The reported samples are the only ones at equilibrium conditions. The clinopyroxene compositions are reported in Supplementary Tab. 6 and the whole rock compositions are from refs. 11, 12 and 13. The error (± 0.2 GPa for pressure and ± 27 °C for temperature) takes into account the uncertainties from both the geothermobarometry model (± 0.1 GPa and ± 27 °C respectively)[9] and the electron microprobe analyses (± 0.1 GPa for pressure only, deriving from the ± 10 % accuracy on measured Na concentration).

Supplementary Tables

Samples	Geographical and geological provenance
AL14	Algarve Basin, basalt lava flow (Portugal)
AN18	Northern Central High Atlas, Intermediate basalt lava flow (Ait Ourir, Morocco)
AN39	Northern Central High Atlas, Intermediate basalt lava flow (Jebel Imizar, Morocco)
AN137A	Southern Central High Atlas, Intermediate basalt lava flow (Tiourjdal, Morocco)
AN156A	Southern Central High Atlas, Recurrent basalt lava flow (Tiourjdal, Morocco)
NEW31	Newark Basin, Orange Mt. basalt lava flow (Clifton UBC Quarry, New Jersey, USA)
NEW73	Newark Basin, Hook Mt. basalt lava flow (New Jersey, USA)
NEW136B	Newark Basin, Palisades sill (Fort Lee, New Jersey, USA)
NS9	Fundy Basin, Upper North Mt. basalt lava flow (Scott's Bay, Nova Scotia, Canada)
NS12	Fundy Basin, Middle North Mt. basalt lava flow (Ross Creek, Nova Scotia, Canada)
NS13	Fundy Basin, Upper North Mt. basalt lava flow (Baxters Harbour, Nova Scotia, Canada)
NS21	Fundy Basin, Upper North Mt. basalt lava flow (Culloden, Nova Scotia, Canada)

Supplementary Table 1 Samples provenance. Geographical and geological provenance of all basaltic samples analysed in this study.

	SiO_2	TiO_2	Al_2O_3	FeO	MnO	MgO	CaO	Na_2O	K_2O	Cr_2O_3	SO_3	Cl	Total
**Milano EMP data**													
AN137A-1	60.62	0.85	21.55	5.50	0.07	0.18	5.61	4.30	1.37	0.09	0.10	0.00	100.24
AN137A-3	60.57	0.75	21.04	5.30	0.03	0.06	5.29	3.99	1.47	0.03	0.03	0.00	98.56
AN137A-5	59.40	0.70	21.32	5.38	0.07	0.48	5.96	4.77	1.25	0.00	0.07	0.01	99.40
AN137A-2	58.33	1.12	20.52	6.33	0.12	0.24	5.06	3.14	1.44	0.00	0.20	0.03	96.53
AN137A-2BIS	59.22	1.12	21.01	6.05	0.08	0.18	5.06	3.30	1.48	0.01	0.10	0.00	97.62
AN137A-4	61.82	0.74	20.68	4.63	0.11	0.09	5.26	4.15	1.56	0.02	0.01	0.01	99.07
NS9-3	58.55	1.39	22.05	6.01	0.02	0.20	5.32	4.81	1.52	0.14	0.14	0.00	100.15
NS9-3BIS	58.79	1.21	21.66	5.57	0.04	0.18	5.39	4.62	1.51	0.01	0.15	0.00	99.12
NS9-2	61.94	1.10	19.33	4.76	0.08	0.15	4.85	4.24	2.10	0.01	0.00	0.00	98.55
NS9-4	59.59	1.26	21.12	5.58	0.07	0.23	5.12	4.41	1.49	0.00	0.12	0.01	99.00
NS9-5	58.53	1.16	20.15	5.89	0.05	0.31	5.56	4.45	1.35	0.00	0.12	0.01	97.57
NS9-5BIS	59.08	1.12	20.72	5.55	0.12	0.36	5.49	4.17	1.50	0.00	0.17	0.01	98.28
NS9-1	60.63	1.05	20.27	5.80	0.07	0.56	5.08	4.91	1.55	0.00	0.17	0.00	100.08
NS9-1BIS	59.80	0.95	21.66	5.16	0.06	0.46	5.02	5.15	1.49	0.00	0.06	0.02	99.83
NS12-5a	57.97	1.19	20.06	5.85	0.12	0.49	5.63	4.12	1.63	0.04	0.10	0.00	97.18
NS12-5b	58.47	0.70	21.20	5.65	0.16	0.43	5.14	4.56	1.69	0.00	0.06	0.00	98.06
NS12-2	59.27	1.05	21.61	6.13	0.10	0.21	5.26	4.32	1.65	0.00	0.04	0.00	99.63
NS12-3b	60.88	0.35	21.10	1.76	0.00	0.09	6.81	5.52	1.53	0.00	0.00	0.00	98.04
NS12-1	56.74	1.21	20.29	7.93	0.11	0.71	6.22	4.23	1.54	0.00	0.10	0.00	99.08
NS12-4	59.41	1.20	20.86	6.38	0.04	0.39	5.27	4.24	1.47	0.00	0.00	0.02	99.28
**Padova EMP data**													
AL14_2PG	68.41	0.25	14.10	1.61	0.05	2.58	6.17	6.08	1.81	n.a.	n.a.	n.a.	101.07
AN18_3PG	53.69	1.17	15.09	9.28	0.15	6.95	9.61	0.45	0.40	n.a.	n.a.	n.a.	96.78
NEW31_S3_4PxS	56.38	0.00	19.51	7.15	0.16	2.61	7.82	1.89	1.06	0.06	n.a.	n.a.	96.65
NEW31_S3_PxMS_2	57.69	1.31	21.59	6.89	0.07	0.35	5.67	2.20	1.39	0.02	n.a.	n.a.	97.17
NEW31_S3_PxMS_3	57.27	1.29	21.19	9.95	0.05	0.31	5.33	2.51	1.59	0.00	n.a.	n.a.	99.50
NEW31_S3_PxMS_4	59.56	1.28	22.18	6.58	0.10	0.30	5.75	2.39	1.36	0.01	n.a.	n.a.	99.52
NS12_1PG1	57.02	1.19	19.37	6.32	0.06	0.63	5.01	4.29	1.63	n.a.	n.a.	n.a.	95.51
NS12_1PG3	59.45	1.24	19.17	6.80	0.11	1.01	5.67	2.76	1.79	n.a.	n.a.	n.a.	98.00
NS12_1PG4	57.66	1.00	20.53	6.39	0.09	0.45	4.86	4.30	1.51	n.a.	n.a.	n.a.	96.81
NS12_1PG5	59.45	1.00	20.56	6.26	0.09	0.40	5.02	1.96	1.63	n.a.	n.a.	n.a.	96.38
NS12_1PG7	58.46	0.97	20.69	6.48	0.09	0.53	4.99	2.00	1.29	n.a.	n.a.	n.a.	95.49
NS12_1PG8	58.63	0.98	20.63	6.53	0.10	0.81	5.09	1.51	1.50	n.a.	n.a.	n.a.	95.78
NS12_2PG2	58.12	0.99	19.91	6.71	0.07	0.60	5.34	4.63	1.49	n.a.	n.a.	n.a.	97.86
NS12_2PG4	57.97	1.07	18.52	7.37	0.08	1.20	5.96	2.86	1.43	n.a.	n.a.	n.a.	96.45
NS12_2PG7	60.56	0.98	18.31	6.64	0.09	0.83	5.89	3.95	1.30	n.a.	n.a.	n.a.	98.55
NS12_2PG8	60.82	0.97	17.91	6.86	0.12	0.90	6.16	2.81	1.38	n.a.	n.a.	n.a.	97.93

Supplementary Table 2 Electron microprobe data (in wt. %) on the glass of exposed MIs. Beam current was 2 nA for Na, Al, Si and K, and 20 nA for all other elements at Padova, while beam current was 5 nA for all analysed elements at Milano. Accelerating voltage was 20 kV at Padova, and 15 kV at Milano. Counting times were 10 s on the peak and background for Na, Al, Si and K, and 10 s on the peak and 5 s background for all other elements at Padova, while counting times were 10 s on the peak and 5 s background for all analysed elements at Milano. n.a. = not analysed.

Sample	Single-bubble MIs	Multi-bubble MIs
AL14	Common (E or CO_2, without C, CO, CH_4, SO_2, H_2S and H_2O)	Very rare (E or CO_2)
AN18	Common (E or CO_2, without C, CO, CH_4, SO_2, H_2S and H_2O)	Rare (E)
AN39	Common (E or C, without CO, CO_2, CH_4, SO_2, H_2S and H_2O)	Very rare (C)
AN137A	Common (E or CO_2, without CO, CH_4, SO_2, H_2S and H_2O)	Common (E or CO_2)
AN156A	Common (E or CO_2, without C, CO, CH_4, SO_2, H_2S and H_2O)	Rare (E)
NEW31	Common (E or C, without CO_2)	Common, also in Pl (C, without CO_2)
NEW73	Common (E)	Rare, also in Pl (E)
NEW136B	Rare, also in Ol (CO_2)	/
NS9	Common (E or CO_2, without C)	Common (E or CO_2, without C, CH_4 and H_2O)
NS12	/	Common (CO_2, without C, CO, CH_4, SO_2, H_2S and H_2O)
NS13	Common (E?)	Common (E or CO_2, without C)
NS21	Common, in Opx (CO_2, without C, CO, CH_4, SO_2, H_2S and H_2O)	Common, in Opx (CO_2, without C, CO, CH_4, SO_2, H_2S and H_2O)

Supplementary Table 3 Confocal Raman microspectroscopy data on bubbles of all studied samples. For each sample, the frequency of bubble-bearing MIs (hosted in clinopyroxene, when not differently specified) and the presence of investigated compounds within bubbles of MIs (specified in brackets). / = absent or not investigated; Ol = olivine; Opx = orthopyroxene; Pl = plagioclase; E = empty; without... = chemical species investigated, but not found.

Sample	Bands position Upper	Lower	Δ cm^{-1}	Calculated ρ g/cm^3
NEW136B	1286.3	1389.0	**102.7**	**0.103**
AL14	1286.1	1388.8	**102.7**	**0.103**
AL14	1286.5	1389.1	**102.6**	**0.070**
AN18	1286.7	1389.3	**102.6**	**0.070**
AN18	1286.4	1389.2	**102.8**	**0.138**
AN18	1286.5	1389.1	**102.6**	**0.070**
AN137A	1286.4	1389.1	**102.7**	**0.103**
AN137A	1286.4	1389.1	**102.7**	**0.103**
AN137A	1286.4	1389.1	**102.7**	**0.103**
AN137A	1286.5	1389.2	**102.7**	**0.103**
AN137A	1286.4	1389.1	**102.7**	**0.103**
AN137A	1286.3	1389.1	**102.8**	**0.138**
AN137A	1286.3	1389.1	**102.8**	**0.138**
AN137A	1286.5	1389.1	**102.6**	**0.070**
AN137A	1286.4	1389.1	**102.7**	**0.103**
AN137A	1286.4	1389.2	**102.8**	**0.138**
AN137A	1286.3	1389.0	**102.7**	**0.103**
AN137A	1286.3	1389.0	**102.7**	**0.103**
AN156A	1286.0	1388.8	**102.8**	**0.138**
AN156A	1286.2	1389.0	**102.8**	**0.138**
NS9	1286.4	1389.1	**102.7**	**0.103**
NS9	1286.5	1389.1	**102.6**	**0.070**
NS9	1286.6	1389.2	**102.6**	**0.070**
NS9	1286.2	1388.8	**102.6**	**0.070**
NS9	1286.2	1388.8	**102.6**	**0.070**
NS9	1286.2	1388.8	**102.6**	**0.070**
NS9	1286.7	1389.3	**102.6**	**0.070**
NS12	1286.3	1388.9	**102.6**	**0.070**
NS12	1286.2	1389.0	**102.8**	**0.138**
NS12	1286.2	1388.8	**102.6**	**0.070**
NS12	1286.2	1388.9	**102.7**	**0.103**
NS12	1286.5	1389.2	**102.7**	**0.103**
NS12	1286.2	1388.8	**102.6**	**0.070**
NS12	1286.2	1388.9	**102.7**	**0.103**
NS12	1286.3	1388.9	**102.6**	**0.070**
NS12	1286.1	1388.8	**102.7**	**0.103**
NS12	1286.1	1388.8	**102.7**	**0.103**
NS12	1286.0	1388.7	**102.7**	**0.103**
NS12	1286.1	1388.8	**102.7**	**0.103**
NS12	1286.1	1388.8	**102.7**	**0.103**
NS12	1286.0	1388.7	**102.7**	**0.103**
NS13	1286.2	1388.9	**102.7**	**0.103**
NS13	1286.1	1388.8	**102.7**	**0.103**
NS13	1286.1	1388.8	**102.7**	**0.103**
NS21	1286.3	1389.0	**102.7**	**0.103**
NS21	1286.5	1389.2	**102.7**	**0.103**
NS21	1286.2	1388.9	**102.7**	**0.103**
NS21	1286.2	1388.9	**102.7**	**0.103**
NS21	1286.2	1388.9	**102.7**	**0.103**
NS21	1286.2	1389.0	**102.8**	**0.138**
NS21	1286.1	1388.7	**102.6**	**0.070**
NS21	1286.3	1388.9	**102.6**	**0.070**
NS21	1286.1	1388.7	**102.6**	**0.070**
NS21	1286.1	1388.8	**102.7**	**0.103**
**Minimum**			**102.6**	**0.070**
**Maximum**			**102.8**	**0.138**

Supplementary Table 4 Analysis of confocal Raman microspectroscopy data on CO_2-bearing bubbles. First column: samples. Second and third columns: position of upper and lower bands of CO_2 respectively. Fourth column: Fermi diad splitting (Δ), obtained by the difference of the two bands of CO_2. Fifth column: density calculated using the densimeter of ref. 24. At the bottom: minimum and maximum values for the Fermi diad splitting and the density. The acquisition of all data was at room temperature.

Sample	SiO_2 (wt. %)	Error on SiO_2 (wt. %)	H_2O (wt. %)	Error on H_2O (wt. %)	CO_2 (ppm)	Error on CO_2 (ppm)
NEW31_1	57.72	1.34	0.52	0.02	87	12
NEW31_2	57.72	1.34	0.54	0.02	72	12
NEW31_3	57.72	1.34	0.55	0.02	56	12
NS21_1	59.61	1.24	0.59	0.02	31	12
NS21_2	59.61	1.24	0.59	0.02	53	12

Supplementary Table 5 NanoSIMS data and relative uncertainties on the glass of exposed MIs. For SiO_2 content, average values measured on other MIs in the same or similar samples were used. For H_2O and CO_2 contents, the standard deviation was taken into account in the calculation of the error.

	SiO_2	TiO_2	Al_2O_3	FeO	MnO	MgO	CaO	Na_2O	K_2O	Cr_2O_3	SO_3	Cl	Total
**Milano EMP data**													
NS9-5_1	52.09	0.42	2.92	8.40	0.15	17.17	18.70	0.24	0.02	0.19	0.00	0.00	100.30
NS9-5_3	52.43	0.38	2.67	8.60	0.14	17.94	17.90	0.16	0.01	0.29	0.05	0.01	100.59
NS9-5_4	51.93	0.70	2.83	8.42	0.15	16.78	18.64	0.23	0.00	0.19	0.00	0.00	99.88
NS12-1_3	52.99	0.30	2.88	8.50	0.16	18.03	17.34	0.17	0.00	0.25	0.00	0.00	100.62
NS12-1_4	52.33	0.44	2.95	8.66	0.19	17.29	17.74	0.21	0.00	0.24	0.00	0.00	100.04
**Padova EMP data**													
AN18-sito3g-cpx-1_1	52.89	0.30	1.99	6.97	0.21	18.63	18.87	0.19	0.00	0.72	n.a.	n.a.	100.77
AN18-sito3g-cpx-1_3	52.92	0.30	2.00	7.10	0.18	18.61	18.81	0.20	0.02	0.78	n.a.	n.a.	100.91
AN18-sito3g-cpx-1_4	53.03	0.32	2.04	7.00	0.21	18.66	18.91	0.20	0.00	0.79	n.a.	n.a.	101.14
AN18-sito3g-cpx-1_6	52.91	0.28	2.00	7.10	0.14	18.42	18.82	0.18	0.02	0.67	n.a.	n.a.	100.54
AN18-sito3g-cpx-1_8	52.78	0.34	2.10	7.13	0.17	18.65	18.23	0.17	0.00	0.85	n.a.	n.a.	100.44
AN18-sito3g-cpx-1_9	52.59	0.32	2.16	7.02	0.21	18.72	18.48	0.17	0.02	0.78	n.a.	n.a.	100.46
AN18-sito3g-cpx-1_10	52.78	0.33	2.12	7.51	0.13	18.35	18.13	0.18	0.03	0.78	n.a.	n.a.	100.33
AN18-sito3g-cpx-1_11	52.70	0.33	2.19	7.40	0.20	18.51	18.43	0.17	0.00	0.76	n.a.	n.a.	100.69
AN18-sito3g-cpx-1_12	52.85	0.30	2.13	7.14	0.15	18.70	18.32	0.20	0.00	0.82	n.a.	n.a.	100.59
AN18-sito3g-cpx-1_13	52.33	0.44	3.03	7.45	0.18	18.47	18.29	0.19	0.01	0.71	n.a.	n.a.	101.09
AN18-sito3g-cpx-1_14	51.45	0.48	3.24	7.49	0.20	18.21	18.62	0.20	0.00	0.76	n.a.	n.a.	100.63
AN18-sito2g-cpx-2_3	52.81	0.30	1.95	7.45	0.18	18.36	18.79	0.16	0.02	0.44	n.a.	n.a.	100.47
AN18-sito2g-cpx-2_4	52.82	0.35	1.91	7.64	0.21	18.52	18.61	0.17	0.00	0.48	n.a.	n.a.	100.70
AN18-sito2g-cpx-2_5	53.15	0.33	2.01	7.36	0.23	18.44	18.62	0.18	0.01	0.52	n.a.	n.a.	100.86
AN18-sito2g-cpx-2_10	53.00	0.30	1.86	7.43	0.23	18.74	18.58	0.16	0.01	0.50	n.a.	n.a.	100.82
NEW31_Traversa1_2	52.10	0.37	3.43	6.64	0.20	19.32	17.48	0.17	0.01	1.10	n.a.	n.a.	100.81
NEW31_Traversa1_5	53.11	0.32	1.80	7.38	0.20	19.11	18.03	0.19	0.01	0.54	n.a.	n.a.	100.67
NEW31_Traversa1_8	53.00	0.25	2.18	7.50	0.17	19.20	17.55	0.14	0.01	0.71	n.a.	n.a.	100.72
NEW31_Traversa2_1	52.00	0.33	2.86	7.37	0.16	19.20	17.13	0.18	0.01	0.95	n.a.	n.a.	100.19
NEW31_Traversa2_7	52.80	0.37	2.15	7.35	0.19	18.67	17.93	0.14	0.00	0.70	n.a.	n.a.	100.32
NEW31_Traversa2_8	52.80	0.34	2.00	7.22	0.18	18.94	17.74	0.19	0.01	0.68	n.a.	n.a.	100.10
NEW31_Spot26	52.59	0.25	2.49	7.58	0.25	19.22	17.26	0.14	0.00	0.57	n.a.	n.a.	100.36
NEW31_Spot28	52.70	0.31	1.97	7.27	0.22	19.21	17.62	0.15	0.00	0.60	n.a.	n.a.	100.04
NEW31_Traversa3_2	52.00	0.38	3.34	6.48	0.14	18.66	18.36	0.19	0.00	1.17	n.a.	n.a.	100.71
NEW31_Traversa3_3	51.95	0.39	3.34	6.35	0.15	18.44	18.61	0.19	0.00	1.08	n.a.	n.a.	100.49
NEW31_Traversa3_4	51.93	0.39	3.40	6.47	0.18	18.74	18.37	0.19	0.01	1.22	n.a.	n.a.	100.91
NEW31_Traversa3_5	51.89	0.38	3.41	6.49	0.19	19.14	17.59	0.24	0.00	1.27	n.a.	n.a.	100.59
NEW31_Traversa3_6	51.91	0.37	3.44	6.47	0.14	18.85	17.97	0.20	0.00	1.26	n.a.	n.a.	100.60
NEW31_Traversa3_8	51.71	0.37	3.45	6.11	0.13	18.47	18.61	0.23	0.00	1.19	n.a.	n.a.	100.27
NEW31_Traversa3_9	51.90	0.35	3.40	6.11	0.15	18.39	18.77	0.23	0.01	1.21	n.a.	n.a.	100.52
NEW31_Traversa3_10	51.97	0.36	3.36	6.30	0.21	18.64	18.13	0.21	0.00	1.09	n.a.	n.a.	100.26
NEW31_Traversa3_11	51.92	0.35	3.49	6.23	0.19	18.60	18.39	0.26	0.01	1.02	n.a.	n.a.	100.47
NEW31_Traversa3_12	51.64	0.39	3.51	6.01	0.10	18.20	19.08	0.21	0.00	1.22	n.a.	n.a.	100.36
NEW31_Traversa3_13	51.89	0.31	3.43	6.01	0.14	18.16	19.25	0.21	0.00	1.11	n.a.	n.a.	100.51

	SiO_2	TiO_2	Al_2O_3	FeO	MnO	MgO	CaO	Na_2O	K_2O	Cr_2O_3	SO_3	Cl	Total
**Padova EMP data**													
NEW31_Traversa3_14	52.01	0.36	3.47	5.98	0.16	18.24	18.85	0.23	0.00	1.17	n.a.	n.a.	100.48
NEW31_Traversa3_15	52.06	0.35	3.39	6.52	0.21	18.90	17.95	0.21	0.01	1.00	n.a.	n.a.	100.60
NEW31_Traversa3_16	52.22	0.32	3.23	6.46	0.24	19.46	17.42	0.22	0.01	1.14	n.a.	n.a.	100.72
NEW31_Traversa3_17	52.39	0.37	3.10	6.63	0.24	19.66	16.93	0.17	0.00	0.91	n.a.	n.a.	100.40
NEW31_Traversa3_18	52.35	0.34	3.20	6.83	0.19	19.67	16.93	0.20	0.00	1.01	n.a.	n.a.	100.72
NEW31_Traversa3_19	51.96	0.35	3.20	6.46	0.23	18.90	17.95	0.23	0.00	1.06	n.a.	n.a.	100.35
NEW31_Traversa3_20	52.40	0.35	3.20	6.42	0.18	19.41	17.23	0.16	0.01	0.85	n.a.	n.a.	100.20
NEW31_Traversa3_21	52.13	0.35	3.32	6.00	0.19	18.36	18.95	0.21	0.00	1.13	n.a.	n.a.	100.64
NEW31_Traversa3_22	51.81	0.37	3.30	5.86	0.16	18.15	19.42	0.21	0.02	1.12	n.a.	n.a.	100.43
NEW31_Traversa3_23	51.83	0.36	3.47	6.08	0.17	18.12	19.32	0.25	0.04	1.16	n.a.	n.a.	100.81
NEW31_Traversa3_25	51.95	0.37	3.40	6.10	0.15	18.53	18.78	0.20	0.02	1.11	n.a.	n.a.	100.61
NEW31_Traversa3_26	51.95	0.36	3.32	6.38	0.17	18.90	18.24	0.24	0.02	1.08	n.a.	n.a.	100.66
NEW31_Traversa3_28	52.00	0.35	3.29	6.39	0.19	18.71	17.90	0.23	0.00	1.02	n.a.	n.a.	100.08
NEW31_Traversa3_29	52.52	0.33	3.18	6.58	0.17	19.62	17.07	0.24	0.01	1.11	n.a.	n.a.	100.83
NEW31_Traversa3_30	51.81	0.37	3.36	6.13	0.17	19.00	18.28	0.22	0.00	1.11	n.a.	n.a.	100.46
NEW31_Traversa3_31	52.06	0.39	3.49	6.20	0.16	18.59	18.49	0.18	0.00	1.12	n.a.	n.a.	100.67
NEW31_Traversa3_33	51.84	0.38	3.45	6.23	0.18	18.39	18.52	0.19	0.01	1.11	n.a.	n.a.	100.31
NEW31_Traversa3_34	51.97	0.37	3.37	6.46	0.19	18.70	18.45	0.24	0.00	0.96	n.a.	n.a.	100.69
NEW31_Traversa3_35	51.97	0.33	3.40	6.28	0.19	18.82	18.27	0.17	0.00	1.08	n.a.	n.a.	100.52
NEW31_Traversa3_36	52.08	0.35	3.28	6.51	0.15	19.06	17.90	0.18	0.00	1.08	n.a.	n.a.	100.58
NEW31_Traversa3_37	51.92	0.35	3.33	6.09	0.21	18.73	18.63	0.21	0.00	1.16	n.a.	n.a.	100.63
NEW31_Traversa3_38	52.04	0.33	3.49	6.86	0.20	18.98	17.56	0.20	0.00	1.08	n.a.	n.a.	100.73
NEW31_Traversa3_39	51.74	0.37	3.44	6.53	0.16	18.71	18.00	0.26	0.00	1.02	n.a.	n.a.	100.21
NEW31_Traversa3_40	51.36	0.40	3.44	6.57	0.18	18.46	18.36	0.20	0.01	1.10	n.a.	n.a.	100.08

Supplementary Table 6 Electron microprobe data (in wt. %) on the host clinopyroxene. Beam current was 20 nA at Padova, and 5 nA at Milano. Accelerating voltage was 20 kV at Padova, and 15 kV at Milano. Counting times were 10 s on the peak and 5 s background at Padova and Milano. n.a. = not analysed.

Supplementary References

[1] Hartley, M. E., Maclennan, J., Edmonds, M. & Thordarson, T. Reconstructing the deep CO_2 degassing behaviour of large basaltic fissure eruptions. *Earth Planet. Sci. Lett.* **393**, 120-131 (2014).

[2] Steele-MacInnis, M., Esposito, R., Moore, L. R. & Hartley, M. E. Heterogeneously entrapped, vapor-rich melt inclusions record pre-eruptive magmatic volatile contents. *Contrib. Mineral. Petrol.* **172**, 18 (2017).

[3] Iacono-Marziano, G. *et al.* Extremely reducing conditions reached during basaltic intrusion in organic matter-bearing sediments. *Earth Planet. Sci. Lett.* **357-358**, 319-326 (2012).

[4] Lowenstern, J. B. Melt Inclusions Come of Age: Volatiles, Volcanoes, and Sorby's Legacy. *Dev. Volcanol.* **5**, 1-21 (2003).

[5] Moore, L. R. *et al.* Bubbles matter: An assessment of the contribution of vapor bubbles to melt inclusion volatile budgets. *Am. Mineral.* **100**, 806-823 (2015).

[6] Eggler, D. H. & Burnham, C. W. Crystallization and Fractionation Trends in the System Andesite-H_2O-CO_2-O_2 at Pressures to 10 Kb. *Geol. Soc. Am. Bull.* **84**, 2517-2532 (1973).

[7] Baker, D. R. & Eggler, D. H. Fractionation paths of Atka (Aleutians) high-alumina basalts: Constraints from phase relations. *J. Volcanol. Geotherm. Res.* **18**, 387-404 (1983).

[8] Caricchi, L., Sheldrake, T. E. & Blundy, J. Modulation of magmatic processes by CO_2 flushing. *Earth Planet. Sci. Lett.* **491**, 160-171 (2018).

[9] Putirka, K., Johnson, M., Kinzler, R., Longhi, J. & Walker, D. Thermobarometry of mafic igneous rocks based on clinopyroxene-liquid equilibria, 0-30 kbar. *Contrib. Mineral. Petrol.* **123**, 92-108 (1996).

[10] Putirka, K. D. Thermometers and Barometers for Volcanic Systems. *Rev. Mineral. Geochem.* **69**, 61-120 (2008).

[11] Callegaro, S. *et al.* Enriched mantle source for the Central Atlantic magmatic province: New supporting evidence from southwestern Europe. *Lithos* **188**, 15-32 (2014).

[12] Merle, R. *et al.* Sr, Nd, Pb and Os Isotope Systematics of CAMP Tholeiites from Eastern North America (ENA): Evidence of a Subduction-enriched Mantle Source. *J. Petrol.* **55**, 133-180 (2014).

[13] Marzoli, A. *et al.* The Central Atlantic Magmatic Province (CAMP) in Morocco. *J. Petrol.* **60**, 945-996 (2019).

[14] Callegaro, S. *et al.* Geochemical Constraints Provided by the Freetown Layered Complex (Sierra Leone) on the Origin of High-Ti Tholeiitic CAMP Magmas. *J. Petrol.* **58**, 1811-1840 (2017).

[15] Cashman, K. V., Sparks, R. S. J. & Blundy, J. D. Vertically extensive and unstable magmatic systems: A unified view of igneous processes. *Science* **355**, 6331 (2017).

[16] Putirka, K. D. Down the Crater: Where Magmas are Stored and Why They Erupt. *Elements* **13**, 11-16 (2017).

[17] Jackson, M. D., Blundy, J. & Sparks, R. S. J. Chemical differentiation, cold storage and remobilization of magma in the Earth's crust. *Nature* **564**, 405-409 (2018).

[18] Papale, P., Moretti, R. & Barbato, D. The compositional dependence of the saturation surface of H_2O+CO_2 fluids in silicate melts. *Chem. Geol.* **229**, 78-95 (2006).

[19] Frezzotti, M. L. Silicate-melt inclusions in magmatic rocks: applications to petrology. *Lithos* **55**, 273-299 (2001).

[20] Audétat, A. & Lowenstern, J. B. Melt Inclusions. Chapter 6 in Geochemistry of Mineral Deposits. *Treatise on Geochemistry* **13**, 143-173 (2014).

[21] Moore, L. R., Mironov, N., Portnyagin, M., Gazel, E. & Bodnar, R. J. Volatile contents of primitive bubble-bearing melt inclusions from Klyuchevskoy volcano, Kamchatka: Comparison of volatile contents determined by mass-balance versus experimental homogenization. *J. Volcanol. Geotherm. Res.* **358**, 124-131 (2018).

[22] Mironov, N. *et al.* Quantification of the CO_2 budget and H_2O-CO_2 systematics in subduction-zone magmas through the experimental hydration of melt inclusions in olivine at high H_2O pressure. *Earth Planet. Sci. Lett.* **425**, 1-11 (2015).

[23] Le Maitre, R. W. Igneous Rocks: A Classification and Glossary of Terms. Recommendations of the International Union of Geological Sciences Subcommission on the Systematics of Igneous Rocks. 236 pp. (Cambridge University Press, Cambridge, 2002).

[24] Kawakami, Y., Yamamoto, J. & Kagi, H. Micro-Raman Densimeter for CO_2 Inclusions in Mantle-Derived Minerals. *Appl. Spectrosc.* **57**, 1333-1339 (2003).
